# Amelioration of high-fat diet (HFD) + CCl4 induced NASH/NAFLD in CF-1 mice by activation of SIRT-1 using cinnamoyl sulfonamide hydroxamate derivatives: in-silico molecular modelling and in-vivo prediction

**DOI:** 10.1007/s13205-022-03192-5

**Published:** 2022-06-15

**Authors:** Nalini Sodum, Vanishree Rao, Sri Pragnya Cheruku, Gautam Kumar, Runali Sankhe, Anoop Kishore, Nitesh Kumar, C. Mallikarjuna Rao

**Affiliations:** 1 Department of Pharmacology, Manipal College of Pharmaceutical Sciences, Manipal Academy of Higher Education, Manipal, 576104 Karnataka India; 2 Department of Pharmacology and Toxicology, National Institute of Pharmaceutical Education and Research (NIPER), Export Promotions Industrial Park (EPIP), Industrial Area Hajipur, Vaishali District, Hajipur, 844102 Bihar India

**Keywords:** NOTCH-1 receptor, SIRT-1, NAFLD/NASH, ADME, Molecular dynamics, Anti-oxidants

## Abstract

Non-alcoholic fatty liver disease (NAFLD) is one of the major hepatic metabolic disorders that occurs because of the accumulation of lipids in hepatocytes in the form of free fatty acids (FFA) and triglycerides (TG) which become non-alcoholic steatohepatitis (NASH). NOTCH-1 receptors act as novel targets for the development of NAFLD/NASH, where overexpression of NOTCH-1 receptor alters the lipid metabolism in hepatocytes leading to NAFLD. SIRT-1 deacetylates the NOTCH-1 receptor and inhibits NAFLD. Hence, computer-aided drug design (CADD) was used to check the SIRT-1 activation ability of cinnamic sulfonyl hydroxamate derivatives (NMJ 1–8), resveratrol, and vorinostat. SIRT-1 (PDB ID: 5BTR) was docked with eight hydroxamate derivatives and vorinostat using Schrödinger software. Based on binding energy obtained (– 26.31 to – 47.34 kcal/mol), vorinostat, NMJ-2, NMJ-3, NMJ-5 were selected for induced-fit docking (IFD) and results were within – 750.70 to – 753.22 kcal/mol. Qikprop tool was used to analyse the pre pharmacokinetic parameters (ADME analysis) of all hydroxamate compounds. As observed in the molecular dynamic (MD) study, NMJ-2, NMJ-3 were showing acceptable results for activation of SIRT-1. Based on these predictions, in-vivo studies were conducted in CF1 mice, where NMJ-3 showed significant (*p* < 0.05) changes in lipid profile and anti-oxidant parameters (Catalase, SOD, GSH, nitrite, and LPO) and plasma insulin levels. NMJ-3 treatment also reduced inflammation, fibrosis, and necrosis in liver samples.

## Introduction

Non-alcoholic steatohepatitis (NASH) is a hepatic metabolic disorder. The severe progression in non-alcoholic fatty liver disease (NAFLD) is responsible for the pathogenesis of NASH. In NAFLD, the excessive lipids are accumulated in hepatocytes in the form of free fatty acids (FFA), and triglycerides (TG), which are responsible for the formation of lipid droplets in hepatocytes and increase liver weight up to > 5% without alcohol consumption. It causes hepatic steatosis, inflammation, and fibrosis finally leading to hepatocellular carcinoma (HCC) in hepatocytes. The imbalance of lipid metabolism occurs from uptake, metabolism, export, and oxidation of fatty acids in hepatocytes (Chiappini et al. 2017; Keute et al. 2019). NAFLD is asymptomatic until the development of cirrhosis and hepatic decompensation. The “two-hit-theory” explain the NAFLD to steatohepatitis, from which first hit corresponds to explain the metabolic disorders like obesity, insulin resistance (IR), and diabetes mellitus. Metabolic disorders are major risk factors for NASH. The second hit corresponds to explain the oxidative stress, (pro-inflammatory cytokines) inflammation, lipid peroxidation, immune system response, gut, adipose tissue-derived factors, and genetic alteration by inducing or due to hepatocyte injury and fibrosis. NASH patients are at high risk for increased aminotransferase levels, so a liver biopsy is the gold standard method for the diagnosis of NASH patients (Vongsuvanh et al. 2012; Ganbold et al. 2019).

Obesity, one of the major risk factors of NAFLD, causes overexpression and activation of the NOTCH receptor pathway. Findings from recent studies suggest that the NOTCH receptor activation increases the risk of NAFLD through the accumulation of FFA and lipids (TG) in hepatocytes. Metabolic disorders like Type-2 diabetes, IR, obesity, and cardiovascular disorders are the main risk factors for NAFLD. NOTCH signalling pathway is one of the major molecular pathways involved in the various biological process including adipogenesis, etc., (Nueda et al. 2018). NOTCH receptors are single-pass transmembrane receptors, involved in cell–cell communications, which are activated by canonical ligands from the jag (jag), delta (dl), and serrate (ser) transmembrane family. These receptors contain three parts in their structure, NOTCH extracellular domain (NECD), heterodimer, and NOTCH intracellular domain (NICD). Extracellular domain-containing extracellular growth factors (up to 29–31) vary from different subtypes of their receptor family. NOTCH receptors are of four types, namely, NOTCH-1, NOTCH-2, NOTCH-3, and NOTCH-4. Among these, NOTCH-1 receptor is mainly expressed in humans and mice, and is mainly involved in the development of the pathogenesis of NAFLD (Andersson et al. 2011; Pandey et al. 2019).

NOTCH intracellular domain regulates the sterol regulatory elementary binding protein (SREBP1c) transcription factor, involved in FFA. Activation of SREBP1c causes lipotoxicity (increases lipid-mediated metabolic stress) in hepatocytes that contribute to the development of obesity, IR, type 2 diabetes, dyslipidemia, NAFLD, and NASH (Davenport et al. 2014; Jeon and Osborne 2012).

Sirtuins are NAD-dependent hydrolases and endogenous biological substances, and are classified into seven types, namely, SIRT-1 to 7. SIRT-1 possesses anti-inflammatory properties, and improves insulin sensitivity and secretion. Recent research studies reported that low levels of SIRT-1 gene expression were observed and involved in the pathogenesis of NAFLD persons (Colak et al. 2011). Resveratrol was shown to activate SIRT-1 under in-vivo settings and SIRT-1 activation led to deacetylation of NOTCH-1 receptor which resulted in inhibition of NOTCH-1 activity (Zhou et al. 2018) (Bai et al. 2018).

**Fig. 1:**
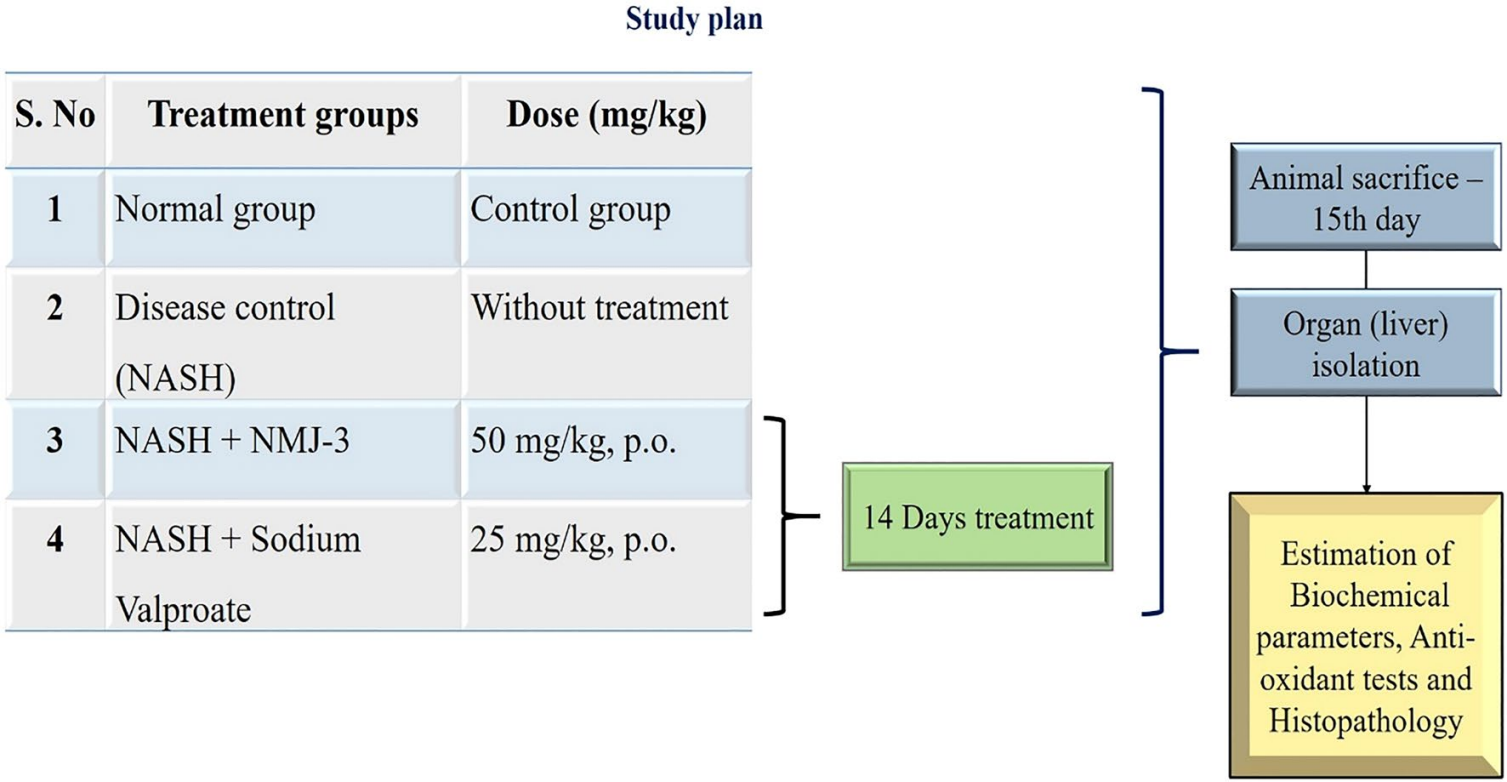
Treatment groups and study plan

The treatment modalities for NAFLD/NASH are very few and associated with several limitations. Hence, in the present study, we hypothesize that SIRT-1-mediated inhibition of the NOTCH-1 receptor can be an effective treatment to overcome the disease progression in NAFLD/NASH. In in-vivo studies, sodium valproate showed potent inhibition of NOTCH-1 receptors by activation of SIRT-1. Novel cinnamoyl sulfonamide hydroxamate derivatives were tested for their inhibitory action. The present study was carried out using different in-silico tools such as molecular docking and molecular dynamics from Schrödinger, maestro, LLC software. All the in-silico (Protein ID: 5BTR) and in-vivo (high-fat diet + CCl_4_ induced NAFLD/NASH in CF-1 mice) experiments were carried out using eight synthesized cinnamyl sulfonamide hydroxamate derivatives to identify their inhibitory action against NOTCH-1 receptors by activation of SIRT-1 and disease prevention activity in NAFLD/NASH animal model (Fig. 1).

## Materials and methods

### *In-silico* molecular modelling of SIRT-1 activation using cinnamoyl sulfonamide hydroxamate derivatives

Software: Schrödinger, LLC using maestro molecular modelling platform (version 10.5); PDB ID: X-ray crystallographic structure was downloaded from the protein data bank (PDB), 5BTR: SIRT-1 protein, resolution: 3.2 A^o^; ligands to be docked: cinnamyl sulfonamide hydroxamate derivatives: NMJ-1, NMJ-2, NMJ-3, NMJ-4 NMJ-5, NMJ-6, NMJ-7, NMJ-8, and Vorinostat.

### Ligand preparation

Synthetic cinnamyl sulphonamide hydroxamate derivatives were selected from Reddy et al. 2015. The Ligprep tool was used to convert 3D structures from the 2D structure and to generate the lowest energies of 3D structures of isomers/tautomers at neutral pH 7.0 ± 2.0 under OPLS3e (optimized potential for liquid stimulation) force field (Sastry et al. 2013; Mallik et al. 2017). The isomers are generated based on the number of chiral carbons present in the chemical structure (ligands-cinnamyl sulphonamide hydroxamate derivatives), based on Le Bel–Van 't Hoff rule 2^*n*^ (*n* = no. of chirality carbons present in chemical structure).

### Protein preparation and grid generation

Protein structure with PDB ID 5BTR corresponding to SIRT-1 was downloaded from the PDB. It is an X-ray crystallographic structure with a 3.2 A^o^ resolution. The protein preparation wizard from Schrodinger software was used to prepare the protein at the lowest energy (Sastry et al. 2013). Multiple tools were used to optimize the protein, the “prime” tool was used to add the missing side chains of selected proteins, and missing residues were updated. The heavy atoms and water molecules were removed, and further optimization of protein was done. The hydrogen bonds were optimized, and energy minimization was done using the OPLS3e force field. After protein preparation, the grid was generated using the Vander Waals scaling factor and charge cut-off under the OPLS3e force field. A cubic box was generated around the selected active site of the protein (Mallik et al. 2017).

### Ligand docking

The ligand docking tool was used to perform the docking studies using the GLIDE module (grid-based ligand docking with energetics) and to predict the ranking and binding modes of the protein–ligand complex. The Ligprep ligands were screened using default settings of extra precision (XP) mode from the GLIDE module and docking score was calculated for each ligand. XP-docking mode was selected as results obtained with this mode are most accurate and incidences of false-positive results are minimum. This is achieved with help of explicit water technology and descriptor that is not found in the other modes like high-throughput virtual screening mode and standard precision docking mode. (Nagpal et al. 2012).

### Prime MM-GBSA free energy calculations (molecular mechanics/generalized born surface area)

MMGBSA assay tool was used to calculate the binding energy of each ligand–receptor complex. The MMGBSA tool from the prime module of the Schrödinger molecular modelling package uses VSGB 2.0 solvation model and OPLS3e force field to stimulate desired interactions and calculate the binding energy (Choudhary et al. 2020). The binding free energy (Δ*G*_bind)_ was calculated using the following formula:

Δ*G*_bind_  =  *E*_complex_ – *E*_protein_ – *E*_ligand_

### Induced fit docking (IFD) standard precision (SP)

Induced fit docking was performed for 8 analogues of cinnamoyl sulfonamide hydroxamate derivatives, which were selected based on the XP-docking score and binding energy. This process allows the conformational changes of ligands to accommodate nearby reorienting side chains. For each ligand, a maximum of 20 poses were generated with each residue falling within 5 A^O^. In IFD, the side chains of the residues are flexible and residues on the backbone remain fixed. Finally, each ligand was re-docked with corresponding low-energy protein structures and the resulting complexes were ranked according to Glide score (Clark et al. 2016). IFD scores for each of these compounds were generated and reported using the formula

IFD Score = 1.0 × Prime_ Energy + 9.057 × Glide Score + 1.428 × Glide_ Ecoul” (Mallik et al. 2017)

### Pharmacokinetic studies (ADME)

Pharmacokinetic parameters play a vital role during drug distribution. Many drugs fail to enter the market due to poor ADME parameters (Shahbazi et al. 2016). Hence, in the present study, QikProp tool from Schrödinger software was used to predict ADME parameters. During this, various descriptors, such as partition coefficient (QlogPo/w), IC50 values for the blockage of HERG k + channel (QPlog HERG), Solubility (QPlogS), Caco-2 cell permeability (QPPCaco), % human oral absorption, and polar surfaces (Vander Waal forces—PSA) of nitrogen, oxygen, and carbonyl carbon atoms indicating the oral bioavailability, and Lipinski’s rule (rule of five) explains suitability of the drug for oral absorption, were calculated (Kumar et al. 2019).

### Molecular dynamic simulations (MD)

Based on docking score, binding energy, residues involved, IFDscore, and ADME analysis, NMJ-2, 3, 5 and vorinostat were selected for molecular simulation studies. The molecular simulation provides flexibility towards the receptor and mimics the realistic scenario of a biological system. Schrodinger, maestro interface, and desmond tool were used for the MD study. This is three-step process, which follows three different panels provided by the maestro interface, namely, system builder (membrane generation—mimicking the cellular membrane), minimization, and molecular dynamic (MD) simulation (drug and membrane allow for dynamic studies). In Desmond, the first tool, i.e., the system builder was used to generate the molecular membrane for the desired protein under the suitable solvent. The total simulation period was set to 100 ns to allow the generation of 1000 frames for dynamic simulation. The protein–ligand complex was selected, and the system model was set to predefined SPC solvent under orthorhombic boundary conditions. In the next step, the minimization tool was used for energy minimization until a gradient threshold reached 25 kcal/mol/Å, balanced at 300 K temperature, and 1 bar pressure via NPT ensemble. Minimization relaxes the system energy into minimum local energy using LBFGS algorithms. In the final step, minimized protein–ligand complex was subjected to MD simulation using a molecular dynamics tool. MD jobs activate the Newtonian dynamics of the model system, generating particle coordinates, energies, and velocities on the model system. RMSD (root-mean-square deviation) was calculated, and it was used to measure the average changes in the position of the selected atoms under the selected frame concerning the standard frame. (Gupta et al. 2019; Volkov and Strizhak 2018).

### Bio-isostere replacement

The top two compounds (NMJ-2, 3) were selected for bioisostere replacement of functional groups, which were used to analyse the optimization of pharmacokinetic parameters and biological properties. Bio-isostere replacement tool from maestro software was used to replace the different functional groups with selected compounds for better potency and pharmacokinetic profiles. Hence, generated bio-isosteres results were analyzed through a ligand interaction with amino acid residues, XP-docking score, binding energy, and ADME analysis (Joel et al. 2020).

### In-vivo analysis for prediction of in-silico analysis

#### Standardization of parameters required for high fat diet-induced NAFLD in mouse

#### Animals

Male CF1 mice (20–25 gm) were procured from the central animal research facility of MAHE, Manipal. The animals were acclimatized at room conditions for (temperature of 23 ± 2 °C and humidity of 50 ± 5 °C) 10 days before starting the experiment. The protocol was framed based on the guidelines provided by the committee for control and supervision of experiments on animals (CPCSEA), Government of India. The experiment protocol was approved by the Institutional animal ethics committee (IAEC), Kasturba Medical College, MAHE (No: IAEC/KMC/107/2019).

### High fat diet (HFD) preparation

HFD was prepared as per our previous in-house study and published paper by (Keni et al. 2020). The diet was composed of fat (58%), protein (25%), and carbohydrate (17%). All the ingredients were weighed (as mentioned in Table 1) and mixed thoroughly. Melted lard was added in the end to obtain the consistency and it was stored at – 20 °C. Here, HFD contains 58% of fat in the form of lard, and this feed composition was described elsewhere (Srinivasan et al. 2005).

**Table 1:** Ingredients used for the preparation of high fat diet (HFD)

S. No	Ingredients	Diet (g/kg)
1	Powdered NPD	365
2	Lard	310
3	Casein	250
4	Cholesterol	10
5	Vitamin and mineral mix	60
6	DL-Methionine	03
7	Yeast powder	01
8	Sodium chloride (NaCl)	01

### Experimental design

#### Disease (NAFLD/NASH) induction

Animals were divided into two groups for inducing NAFLD, a normal pellet diet was given to the normal control (NC) group until the end of the study, and HFD was given to the disease induction group until the end of the study. Every week, body weight was measured, and TG, TC, AST, and ALT parameters were assessed every two weeks. At end of the 8th week, lipid parameters of the HFD group significantly increased and stabilized when compared to the NC group. Subsequently, first and second doses of 0.05 ml/kg of CCl_4_ were given at 8th and 10th week, respectively, to induce hepatotoxicity. CCl_4_ was dissolved in olive oil (vehicle) and was administered intraperitoneally (IP) based on body weight. In hepatocytes, CCl_4_ is converted to CCl_3_ free radicles, which interacts with unsaturated lipids and damages the cell membrane integrity. It increases hepatic lipid profile, inflammatory enzymes, and oxidative stress. CCl_4_ is primarily given to induce liver damage, but the damage does not resemble NAFLD. Therefore, a combination of HFD and CCl_4_ was administered as it increases oxidative stress, initiates hepatic inflammation causes fibrosis, and induces NAFLD/NASH. No death was observed at this dose of CCl_4_. Biochemical parameters were tested in the 11th week for estimation of disease markers like TG, TC, AST, ALT, ASP, and glucose. In CCl_4_-treated group, 40% mortality was observed after the first dose. Later, the mortality was observed to reduce.

### Treatment: preparation and administration of drugs

Disease-induced animals were divided into three groups, with six animals in each group. Treatment drug NMJ-3 (50 mg/kg) and standard drug sodium valproate (25 mg/kg) were prepared using CMC as a solvent. Drugs were administered to treatment groups for 14 days along with HFD. At the end of the treatment, again lipid profile parameters such as AST, ALT, ALP, TG, TC, and glucose were tested. After 14 days of treatment, animals were sacrificed and the liver was isolated for further anti-oxidant and histological examination.

### Anti-oxidant parameters

Anti-oxidant parameters like catalase, superoxide dismutase (SOD), GSH lipid peroxidation, and total protein were estimated in the liver tissue (Pathak et al. 2020), using routine laboratory UV spectroscopic and calorimetric methods.

### Biochemical estimation

Blood sample was collected in EDTA (10%) containing tubes from mice by retro-orbital plexus puncture. Subsequently, plasma was separated after centrifuging at 8000 rpm for 10 min at 4 °C temperature. Furthermore, a fully automated analyser (Agappe Diagnostics Ltd, Cochin India) was used to estimate the lipid parameters (AST, ALT, HDL, and LDL). On the other hand, an ELISA microplate reader was used to estimate the total cholesterol (TC), triglycerides (TG), and glucose levels at 490 nm.

### Plasma insulin estimation

Plasma samples collected from the animals were used for insulin estimation by kit assay method.

### Histopathology of liver

Histopathological study was conducted for liver tissue, as the virtue of NAFLD mainly affects liver histology. The tissue was processed by xylene and gradient alcohol with the use of eosin–hematoxylin stain (Ramalingayya et al. 2017).

### Statistical analysis

Graph pad prism (version 7) was used for statistical analysis and all data are expressed as mean ± SEM. All data were analysed by one-way ANOVA using Tukey’s post hoc test, and the *p* < 0.05 value was considered a significant value.

### Results

#### Activation of SIRT-1 using cinnamyl sulfonamide hydroxamate derivatives

In the present study, the protein was prepared under neutral pH = 7.0 ± 2.0, as per the previous studies, reported for properties and activation of 5BTR along with Sirt-1 activator resveratrol, a unique p53-7-amino-4-methyl coumarin peptide is also present. The Sirt-1-143CS protein complex with resveratrol consists of the N-terminal domain (NTD) and the catalytic domain (CD). The NTD of the SIRT-1 possesses the core of three helices (amino acids:184–229). N-terminal domain (NTD) and the catalytic domain (CD) are covalently bonded, with hydrogen bonds GLU230, ARG446.

### Resveratrol binding

Resveratrol binds with SIRT-1 and contains 3 resveratrol molecules Res1, Res2, and Res3. The Res1, Res2 showed hydrogen-bond interaction with SIRT-1 NTD, P53-AMC. Res1 contains two hydroxyl groups in their phenyl moiety structure. One of the hydroxyl groups makes a bond with the Glu230 side chain and another hydroxyl group makes a bond with the amine and carbonyl group of Lys3 of P53-AMC. The Glu230 of Res1 also interacts with Arg446 in the catalytic domain. Res2 is located ~ 4–5 A^O^ away from the Res1, and it has one hydroxyl group in phenyl moiety structure, that interacts with Gln222 and Asn 226 (hydrogen bonds) in SIRT-1 NTD and also with the carbonyl group of Arg1 peptide. Res3 is located opposite to Res1, res2, and nearer to the SIRT-1 CD. The ring structure is dihydroxy phenyl moiety, and it is bound with ASP292 and Asp298 in the catalytic domain (CD). The distance hydroxyl group of phenyl moiety bonded with Lys444.

Based on these results, the interaction of selected hydroxamate derivatives with SIRT-1 (PDB ID:5BTR) was analysed. Few crucial interactions were reported at Asp292, Asp298, and Lys444 amino acid residues previously. In accordance with previously published articles, Chain A of 5BTR was selected for docking and simulation studies.

### Ligand docking

Synthetic eight cinnamyl sulfonamide hydroxamate derivatives were selected from the Reddy et al. (2015). After ligand preparation, the generated isomers/tautomer were docked with 5BTR protein representing the SIRT-1 gene. All eight cinnamyl sulphonamide hydroxamate derivatives showed docking scores in the range of – 2.2 to – 5.5 and the docking score of vorinostat was – 3.2 (Table 2). This explained the formation of possible interactions between ligand and protein (Table 3).

**Table 2:**
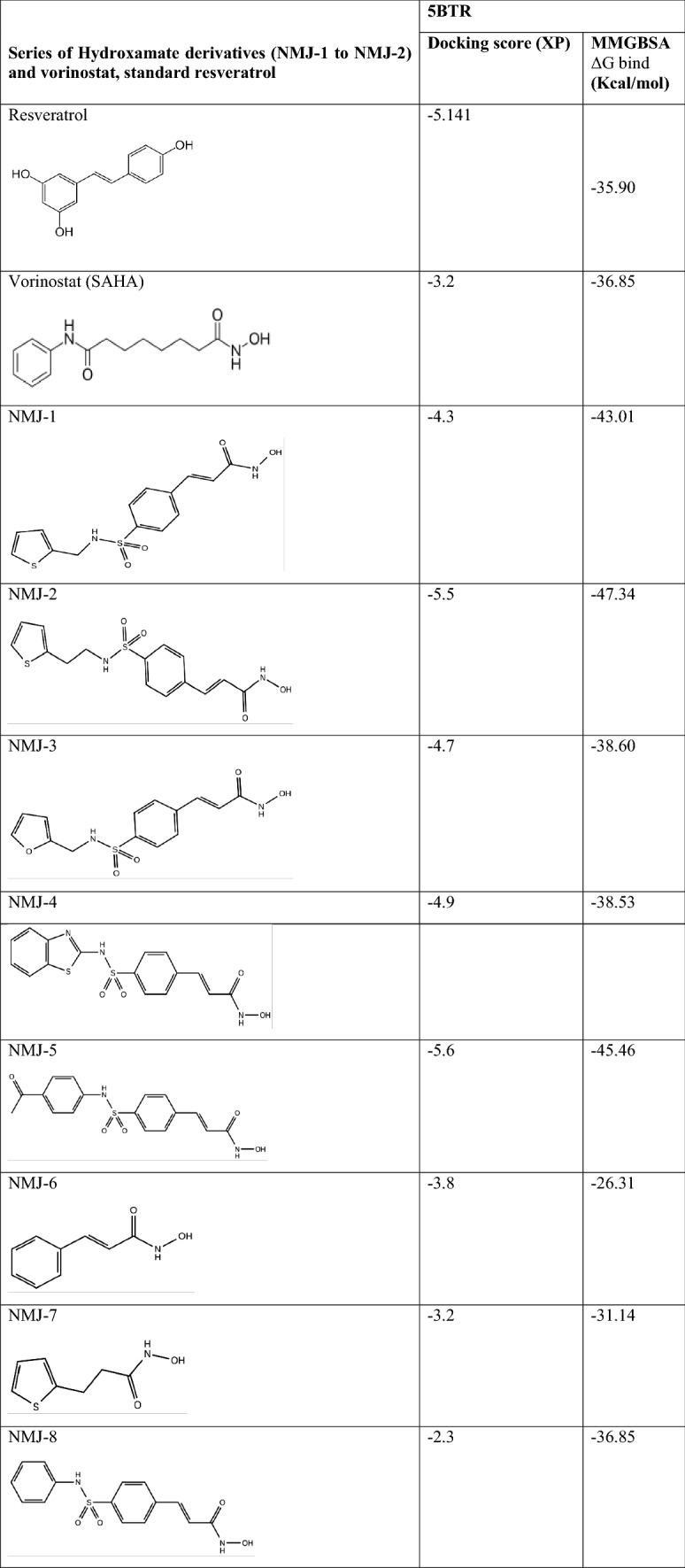
Docking score and binding energy of cinnamoyl sulfonamide hydroxamate derivatives

**Table 3:**
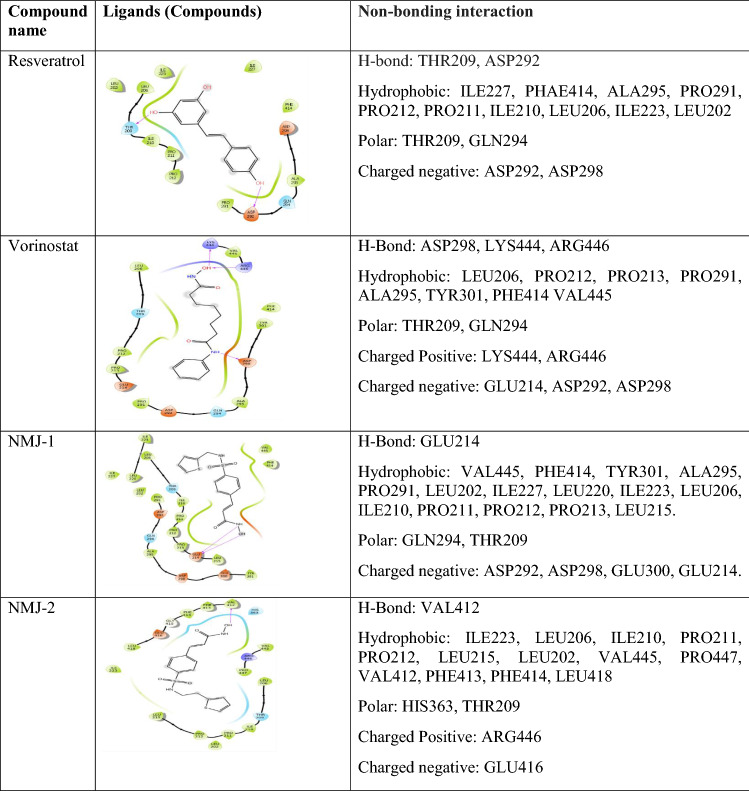

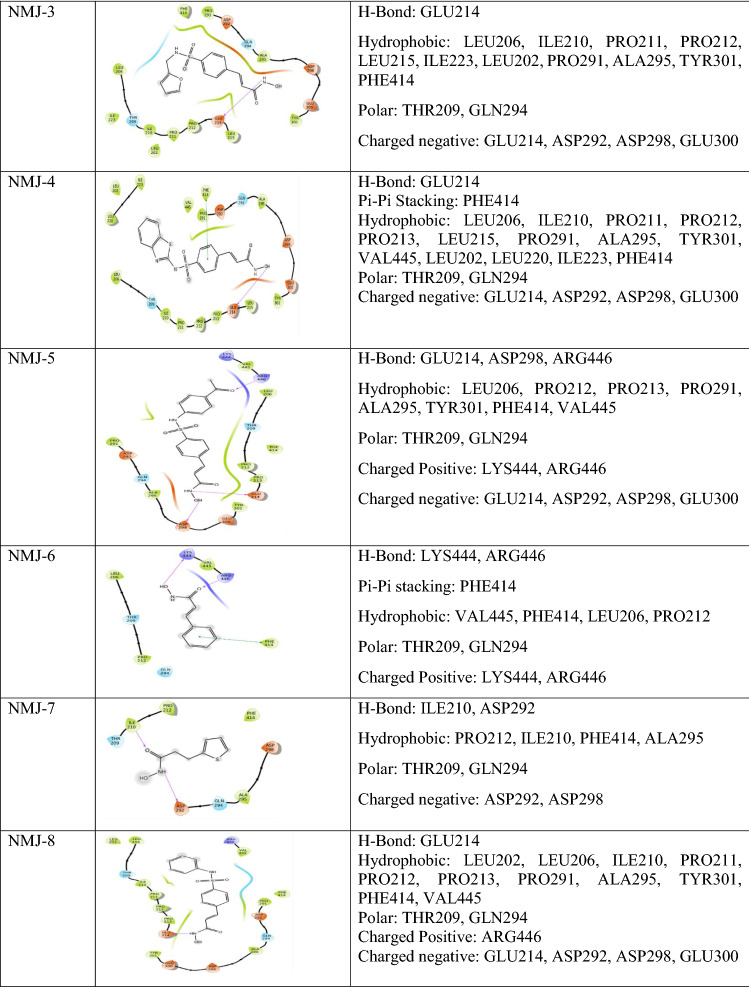
Non-bonding interactions (docking interactions) of vorinostat and ligands (cinnamyl sulfonamide hydroxamate derivatives 1-8) with 5BTR Protein

### Comparative ligand Interactions of hydroxamate derivatives with Res3, Res2, and Res1

After performing docking studies, the ligand interactions of hydroxamate derivatives were compared with Res3. All the hydroxamate derivatives and vorinostat showed negatively charged amino acid interaction with Asp292 and Asp298, except NMJ-2. Out of which, Asp292 formed a hydrogen bond with -NH group of NMJ-7, and Asp298 has formed a hydrogen bond with -OH group of NMJ-5. Lys444 is a positively charged amino acid presented in NMJ-5 and vorinostat. Lys444 has formed a hydrogen bond towards the -OH group of the side chain of vorinostat.

Res1 showed interaction with GLU230, ARG446 similarly, ARG446 (charged positive amino acid), formed interactions with NMJ-2, NMJ-5, NMJ-8, and vorinostat. Arg446 interacted with the carbonyl group of phenyl moiety of NMJ-5 and -OH group of the main side chain of vorinostat.

Literature states that ASP292, ASP298, and LYS444 are important residues involved in resveratrol-mediated activation of SIRT-1. Similar amino acids were also present in vorinostat and hydroxamate derivatives NMJ-1 to 8 ligand interactions. Other than this, interactions with Asp292 and Asp298 were also present in all ligand–protein complexes of hydroxamate derivatives and vorinostat. However, interaction with LYS444 was only present in NMJ-5.

### Binding energy

The binding energy of hydroxamate derivatives was predicted by the prime-MMGBSA tool. It calculated the strength of the compound bound with the target, which explains the stability of the ligand interactions towards the best site of the target. All hydroxamate derivatives showed good ΔG binding energy between – 31 and – 48 kcal/mol (Described in Table 2).

### Induced fit docking-standard precision (IFD-SP) results of selected compounds

Based on the docking score, ligand interaction, and ΔG binding energy, three derivatives, namely, NMJ-2, NMJ-3, NMJ-5, and vorinostat were selected for IFD-SP docking. Results were found to be > 750 kcal/mol (Table 4). During induced-fit docking, up to 20 conformers were generated for each derivative and vorinostat. Each conformer has an individual IFD-SP score. In IFD-SP, new interactions were generated and/or lost as per previous interactions of XP docking. Whereas some ligand interactions were retained after the IFD-SP docking studies. IFD results of Vorinostat revealed that interactions with LYS444, ARG446 (hydrogen bonds) were lost, new interactions such as ILE210, THR209, ASP292 were formed, and interaction with ASP298 was retained as in XP docking. Hydrophobic bonds at LEU206 and PRO212 were present in XP-docking interactions, but they were lost in induced-fit docking interactions and these interactions were replaced by lEU290 and ILE210. PRO291, ALA295, TYR301, PHE414, PRO212, and PRO213 were presented in both XP & IFD-SP. The polar amino acids THR209, GLN294 were in the XP and IFD-SP docking. XP-docking interactions of positively charged amino acids LYS444, ARG446 were replaced by LYS304 during IFD-SP. Negatively charged amino acids GLU214, ASP292, ASP298 were presented in XP and IFD-SP docking, but here one new ligand interaction ASP289 was generated during IFD-SP (described in Table 4).

**Table 4:**
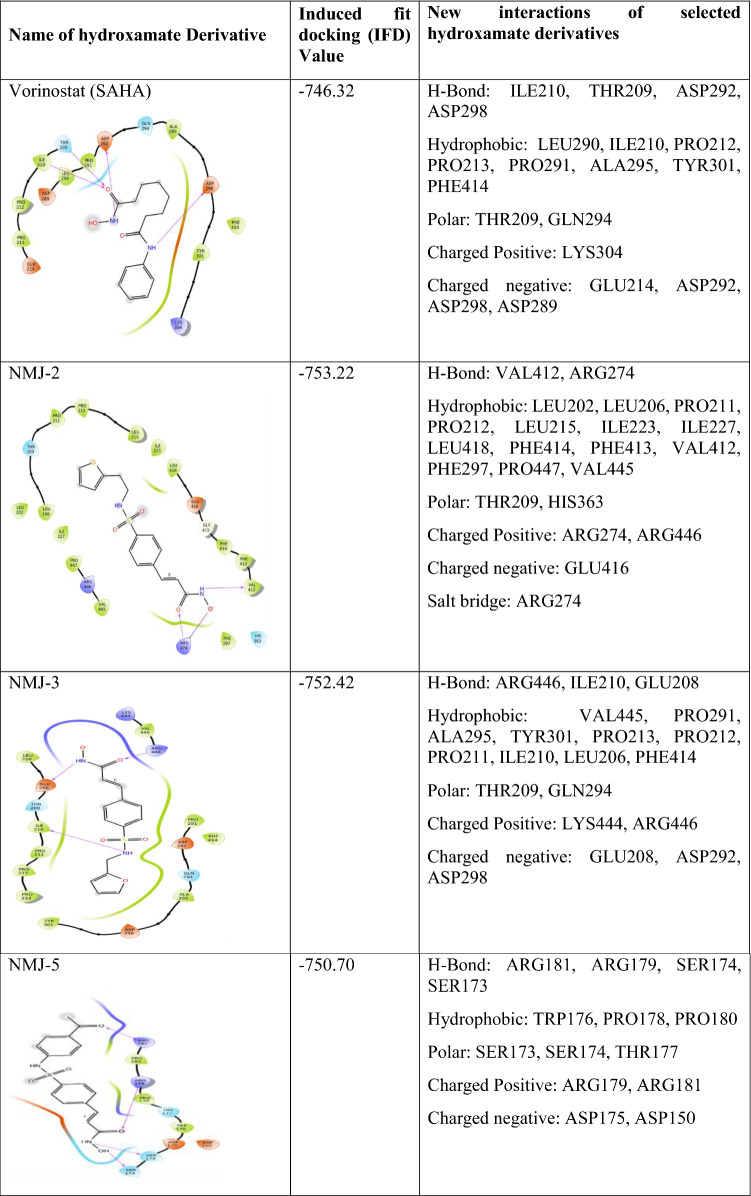
Induced fit docking (IFD-SP) results of selected cinnamyl sulfonamide hydroxamate derivatives and Vorinostat with new interactions

Analysis of hydroxamate derivative (NMJ-2) induced new interactions and/or lost the interactions during IFD-SP compared to interactions of XP docking. The NMJ-2 showed new hydrogen-bond interaction with ARG274 and VAL412 was present in both XP and IFD-SP docking. The hydrophobic contacts like ILE227 and PHE297 were newly generated, whereas the interactions with ILE210. LEU202, LEU206, PRO211, PRO212, LEU215, ILE223, LEU418, PHE414, PHE413, VAL412, PRO447, and VAL445 were lost and polar interactions like THR209, HIS363 were present in both XP-docking and IFD-SP. New positive charged interaction ARG274 was formed and ARG446 was retained. There was no change in the replacement of negatively charged interaction like GLU416. Here, ARG274 made a new electrostatic interaction, i.e., salt bridge, which connected towards the oxygen atom of hydroxyl (–OH) of the ligand side chain (Described in Table 4).

Prediction of NMJ-3 induced-fit ligand contacts was compared with XP-docking interactions. The NMJ-2 showed completely new H-bond interactions with ARG446, ILE210, GLU208, hydrophobic interactions with VAL445, PRO213, positively charged interactions with LYS444, ARG446, and negatively charged interaction with GLU208. The NMJ-3 lost the hydrogen-bond interaction with GLU214, hydrophobic bond interaction with LEU215, ILE223, LEU202, and negatively charged interactions GLN214, GLU300 during IFD-SP. Hydrogen bonds LEU206, ILE210, PRO211, PRO212, PRO291, ALA295, TYR301, PHE414, and polar interactions THR209, GLN294 were present in both XP and IFD-SP (Table 4).

NMJ-5 showed different interaction patterns as compared to XP docking. The NMJ-5 showed hydrogen-bond interaction with Glu214, Asp298, Arg446, hydrophobic bonds with Leu206, Pro212, Pro213, Pro291, Ala295, Tyr301, Phe414, Val445, polarity interaction with Thr209, Gln294, positive charged amino acids interaction with Lys444, Arg446, and negatively charged amino acids interaction with Glu214, Asp292, Asp298, and Glu300. Instead of these interactions, new interactions were generated during induced-fit docking, which includes hydrogen-bond interaction with Arg181, Arg179, Ser174, and Ser173, hydrophobic-bond interaction with Trp176, Pro178, Pro180, polarity amino acids with amino acid Ser173, Ser174, Thr177, positively charged interaction with amino acid residues Arg179, Arg181, and negative amino acids Asp175, Asp150 (Table 4).

### Pharmacokinetic results of cinnamyl sulfonamide hydroxamate derivatives

The pharmacokinetic profile (ADME) of cinnamyl sulphonamide hydroxamate derivatives was predicted using the QikProp tool. ADME parameters of all recommended values are mentioned in Table 5, which explains that compounds containing this range of values have no toxicity. Those compounds that do not obey the recommended values might be causing toxicity.

**Table 5:** Prediction of pharmacokinetic values (ADME) of NMJ series and vorinostat using QIKPROP tool

Compound Id	Molecular weight (MW)	QlogP_o/w_	QlogHERG	QPlogS (mol.dm^−3^	QPPCaco	QPlogBB	% Human oral absorption	PSA	Rule of five (RO5)
Range or recommended values	130.0–725.0	2.0–6.5	< – 5	– 6.5–0.5	< 25 Poor > 500 high	– 3.0 to 1.2 (inactive to active)	> 80% high < 25% poor	7.0–200.0	Max 4
Vorinostat (SAHA)	264.3	0.802	– 4.159	– 2.018	167.438	– 1.604	71.442	99.124	0
NMJ-1	338.396	0.766	– 5.809	– 3.145	67.374	– 2.107	64.154	116.732	0
NMJ-2	352.422	1.054	– 5.889	– 3.343	72.599	– 2.166	66.424	115.266	0
NMJ-3	322.335	0.348	– 5.897	– 2.686	78.341	– 2.122	62.884	124.651	0
NMJ-4	375.03	0.754	– 6.489	– 3.651	74.291	– 2.101	64.848	126.647	0
NMJ-5	360.384	0.172	– 6.219	– 3.317	30.562	– 2.721	54.537	144.141	0
NMJ-6	163.17	0.557	– 4.362	– 1.415	488.459	– 0.834	78.333	65.898	0
NMJ-7	169.2	0.671	– 2.153	– 0.182	287.496	– 0.631	70.437	65.718	0
NMJ-8	318.347	0.67	– 6.223	– 3.026	97.599	– 1.985	66.483	115.136	0

Pharmacokinetic (ADME) values of cinnamyl sulfonamide hydroxamate derivatives along with vorinostat results were in ranges compared with normal values. Molecular weight 163–365 (gm) and partition coefficient QlogP_o/w_ (octanol/water) values showed in the range of 0.1–1.05, which intern means that all the hydroxamate derivatives have good distribution within the body. QPlogHERG for vorinostat, NMJ-6, NMJ-7 was between – 2 and – 6, which indicated that these derivatives may produce cardiac toxicity by blocking of K^+^ channel, but remaining derivatives showed desired QPlogHERG values. These findings also highlighted that the synthesized drugs can be less toxic than vorinostat in terms of cardiotoxicity. The drugs showed solubility results QPlogS in the range of – 3.6 to – 0.182, which explained that all the hydroxamate derivatives have good solubility in the circulation. The range of QPPCaco was 30 to 488.45, which explained that the hydroxamate derivatives have good permeability to cross the cell membrane. The QPlogBB was within – 2.1 to – 0.6, which indicates that the blood–brain barrier permeability will be lesser for cinnamyl sulfonamide hydroxamate derivatives. The percentage (%) of human oral absorption was within 54 to 78, which means that hydroxamate derivatives may get moderately absorbed through the oral route. The PSA (oral bioavailability) of hydroxamate derivatives ranges from 65 to 144 values, explaining that the hydroxamate derivatives have good bioavailability within the circulation. Similarly, all hydroxamate derivatives accepted Lipinski’s rule of five (RO5), so all ligands have acceptable oral active with possible absorption.

### Molecular dynamics (MD) simulation results

Based on docking score, ligand interaction, binding energy, IFD-SP, the NMJ-2, NMJ-3, NMJ-5, and standard vorinostat were selected for molecular dynamics. In molecular dynamics, the selected ligand–protein complexes were exposed to the artificially developed biological system to stimulate the atoms present in the protein–ligand complex. XP & IFD-SP provide only exiguous flexibility, these results were sketchy to mimic the complete schema of the biological system. To understand the stability of selected hydroxamate derivatives with 5BTR protein, MD studies were performed. In MD, protein–ligand complexes were exposed for 100 ns and 1000 frames were generated for calculating the total energies and Vander wall forces. Each frame was captured during simulation every 20 ps and saved in trajectory. The “root mean square deviation” (RMSD) and “Lig Fit Prot” was used for the computation of alpha carbons of SIRT-1 protein and ligand respectively.

**Fig. 2:**
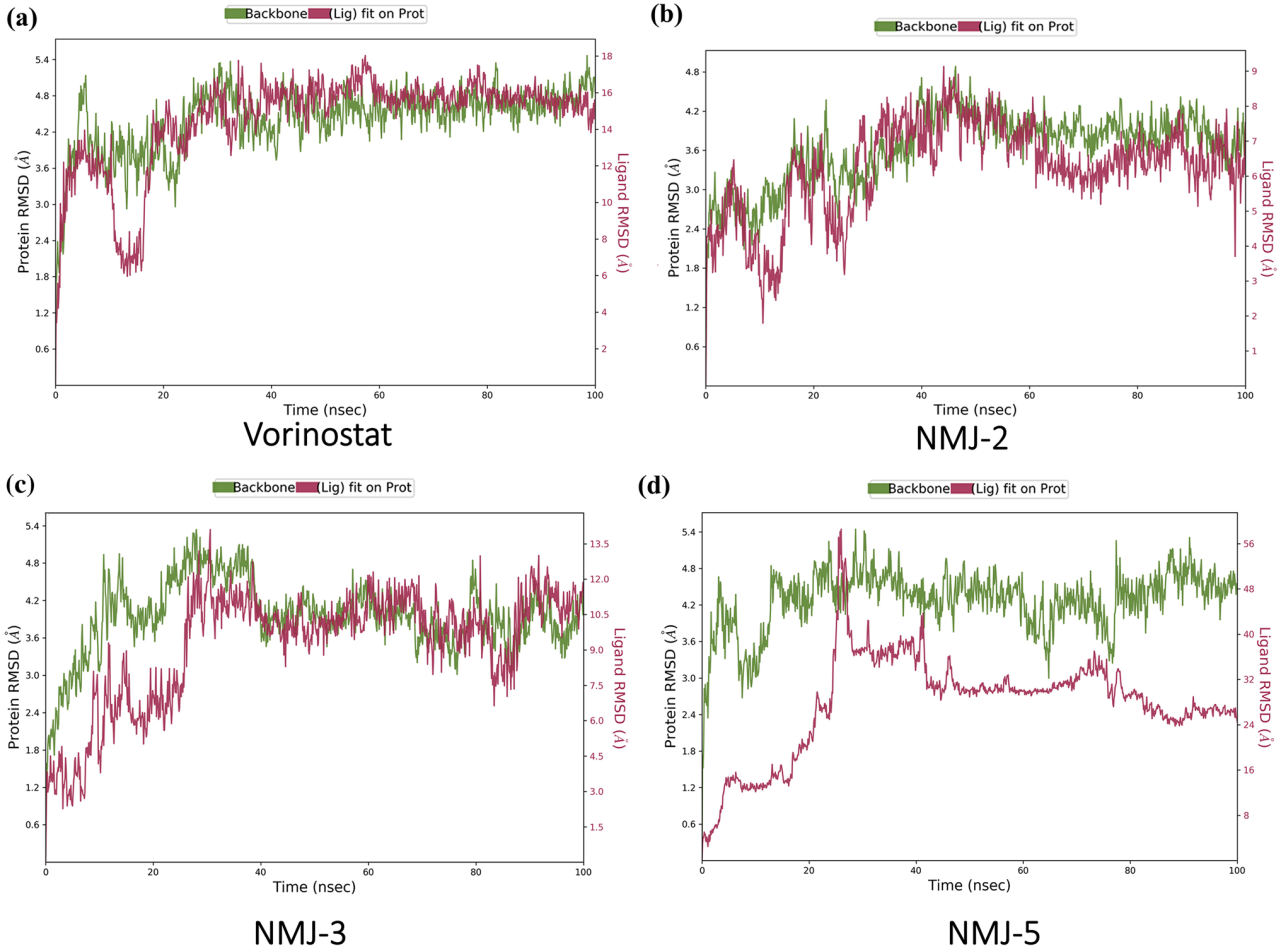
Representation of molecular dynamic (MD) simulation of Protein–Ligand complex root-mean-square deviation (RMSD) of **a** Vorinostat, **b** NMJ-2, **c** NMJ-3, and **d** NMJ-5

The protein–ligand complex of 5BTR-vorinostat (Complex-1) showed an RMSD value of 16Ao and ~ 15 A^o^, respectively. The vorinostat–5BTR complex RMSD value when compared with the reference range, it seemed that the ligand diffused away from the protein in between 0 and 20 ns. Protein–ligand interactions were noticed from 20 to 100 ns, but complex stabilization was not observed. For NMJ-2-5BTR simulation (complex-2), RMSD values were found to be 6Ao and ~ 7Ao, respectively, and the ligand diffused away from protein in the range of 0–20 ns and the 60-80 ns of trajectory period, and the complex was not stabilized. In NMJ-3 with 5BTR complex, the complex-3 showed RMSD value ~ 11 A^o^ and ~ 11 A^o^, respectively, the complex was stabilized after 40 ns to 100 ns. Ligand diffused away from the protein in between 0 and 40 ns. On the other hand, the 5BTR-NMJ-5 (complex-4) of RMSD values are 48, 24 A^o^, respectively, it represents the ligand NMJ-5 completely diffused away from protein in 0–100 ns of trajectory period (Fig. 2).

All non-bonding interactions of four complexes obtained from MD simulation were compared with XP-docking and IFD-SP. In the MD simulation of vorinostat (ligand)–protein interaction, diagram was analysed for the prediction of interactions as compared to XP-docking interactions, only one charged positive ARG446 residue was bound to a ligand with 80% simulation time. During MD simulation, the vorinostat lost all XP-docking interactions except ARG446 and IFD-SP docking interactions. New protein residue ILE223 was observed but without any interactions. Vorinostat lost the XP-docking contacts including H-bond interaction with ASP298, LYS444, hydrophobic contacts with LEU206, PRO212, PRO213, PRO291, TYR301, PHE414, VAL445, polar interaction with amino acids THR209, GLN294, positively charged interaction with amino acids LYS444, and negatively charged interaction with amino acid GLU214, ASP292, ASP298.

According to protein–ligand contact (histogram), the hydrogen bond of ARG446 and SER453 maintained ~ 80% and ~ 40% (~ 0.8 and ~ 0.4) of simulation time, respectively. GLN222, ASN226, ILE227, SER229, ASN417, ALA449, and SER454 residues maintained most of the simulation time with hydrogen bonds, and these residues were also expressed with water bridges. LEU206, PRO212, LEU215, ILE225, and PRO447 formed hydrophobic bonds towards the ligand.

MD simulation of NMJ-2 was analysed for the prediction of interactions as compared to XP-docking and IFD-SP interactions. During the molecular simulation, new interactions were formed along with one water bridge. Water bridge was formed by two hydrogen bonds towards polar charged GLN294 of protein and oxygen atom of the sulfonyl group of ligand and showed 49% simulations for the 100 ns trajectory period. During the molecular dynamic simulation, NMJ-2 formed hydrogen bond interaction with amino acids ASP298, ARG446, GLN294, and THR209. ASP298 made two H-bonds towards the hydroxyl group and amine (–NH) group of a ligand having 39 and 31% simulations, respectively. Hydrophobic amino acid residue PHE414 interacted with the ligand by pi–pi stacking with 31% of simulation time. PHE414, ARG446, and THR209 residues were also present in XP-docking and IFD-SP. New interactions such as negatively charged amino acid ASP298 and polar amino acid GLN294 were observed. As per the protein–ligand contacts (histogram), GLU214 and ASP298 showed ionic interaction fractions along with hydrogen bonds and water bridges. Hydrogen bonds of THR209, ASP298, and ARG446 residues maintained ~ 80% (~ 0.8) of simulation time, hydrophobic contacts were maintained by PHE414. LEU206, PRO211, PRO212, ILE223, ILE227, and ARG446 contained hydrophobic bonds. GLU214, GLN294, and ASP298 protein residues were connected by water bridges towards the ligand. LEU202, GLU208, PRO213, LEU215, ASP289, PRO291, HIS363, GLY415, GLU416, and ALA449 maintained lesser simulation time with their interactions.

Molecular dynamic (MD) simulation of NMJ-3 results was analysed in 100 ns of trajectory period. The ligand was not bound to the protein up to ~ 40 ns, and ligand–protein interaction was observed from 40 to 100 ns. The negatively charged amino acid residues ASP298, GLU214, and polar residue TH209 were bound to the ligand with 35, 53, and 48% binding energy, respectively. Hydrophobic residue ILE210 was observed with 44% binding energy towards the ligand. These all were present in XP-docking and IFD-SP docking, but GLU214 was not observed in IFD but retained at MD simulation. Hydrogen bonds of all amino acid residues were observed in the selected trajectory period. All XP- docking hydrophobic contacts with amino acid residues such as LEU206, PRO211, PRO212, LEU215, ILE223, LEU202, PRO291, ALA295, TYR301, and PHE414, Polar amino acid GLN294, negative charged amino acids were ASP292, and GLU300 were lost, whereas interaction with GLU214 was retained.

According to protein–ligand contact, the hydrogen bond of GLU214 maintained simulation time of ~ 90% (~ 0.9), and THR209, ILE210, GLN294, ASP298, GLY415, and ASN417 simulated by hydrogen bonds along with water bridges. ILU206, PRO212, PRO213, LEU215, ILE223, ILE227, and PHE414 maintained hydrophobic bonds during 100 ns of trajectory period. Protein interactions were not observed from LEU202, PRO211, ARG303, and LEU418.

**Fig. 3:**
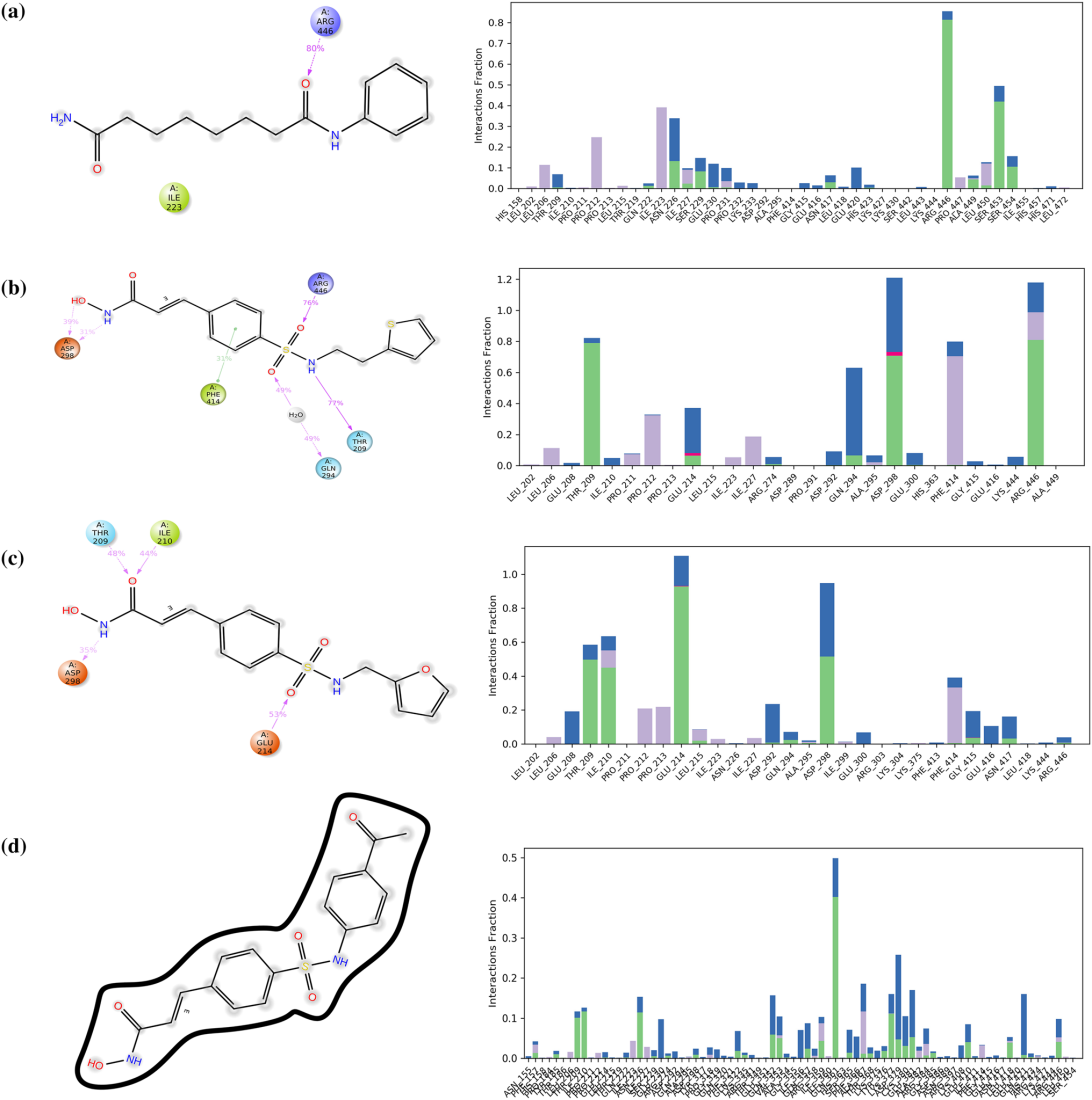
Molecular dynamics (MD) simulated newer ligand–protein contacts and protein residues involved in the formation of interactions (bar diagram). **a** Vorinostat contacts during MD simulation and residues sequence; **b** Hydroxamate derivative (NMJ-2) contacts during MD simulation and residues sequence with acceptable hydrogen bonds, hydrophobic contacts, water bridges, and ionic bonds; **c** Hydroxamate derivative (NMJ-3) contacts during MD simulation and residues sequence with acceptable hydrogen bonds, hydrophobic contacts, water bridges, and ionic bonds. **d** Interactions of NMJ-5 during MD simulation and residues sequence including H-bonds, hydrophobic bonds, ionic bonds, and water bridge

The molecular dynamics study of NMJ-5 was analysed and RMSD (root-mean-square deviation) value was found to be 24 A^o^ (Fig. 3). Here ligand completely diffused from the protein within 100 ns of trajectory period. The system did not reach the equilibrium state, and thus, it is not suitable for rigorous analysis. During MD simulation, NMJ-5 ligand completely lost all XP-docking interactions and IFD ligand contacts. As per the protein–ligand contact (histogram), most of the amino acid residues-maintained hydrogen bonds and water bridges.

### Bio-isostere replacement

**Fig. 4:**
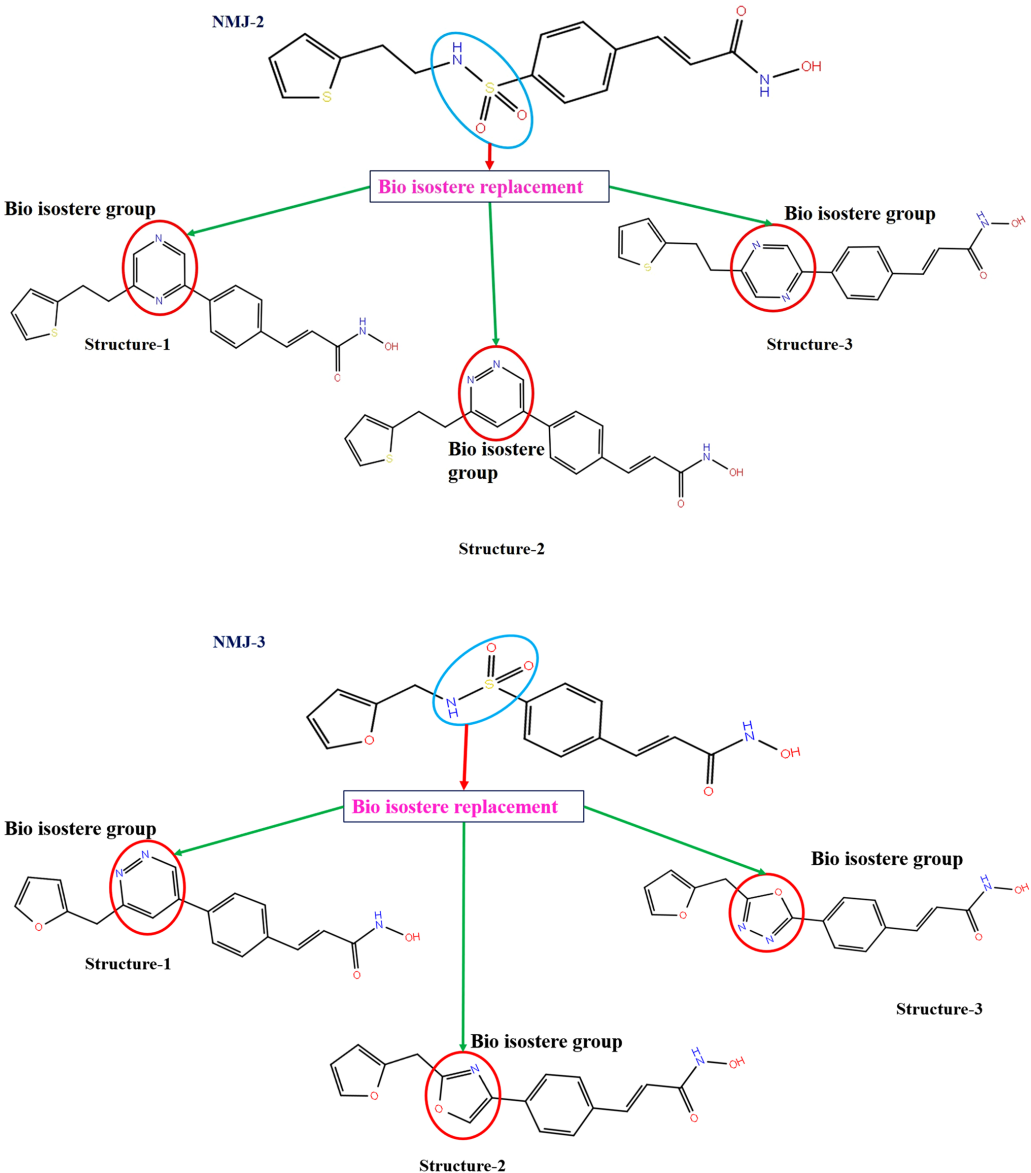
Bioisostereric replacement structures of NMJ-2 and NMJ-3

Based on MD stimulation, NMJ-2 and NMJ-3 were selected for further analysis of bio-isosteric replacement. Here, each compound generated 47 bio-isosteric structures. All structures were allowed to dock with protein, and based on the docking score, the top 3 hits were selected from each derivative. Bio-isosteric structures of NMJ-2 (structure 1, 2 and 3) docking scores were – 5.41, – 5.36, and – 5.473, respectively. Similarly, the docking score of the top 3 hits of NMJ-3 derivative values were – 5.876, – 5.426, and – 5.044, respectively. On the other hand, the binding energy of bio-isosteres of NMJ-2 were – 58.277, – 57.12, and – 56.98 kcal/mol, while NMJ-3 were – 53.12, – 52.81, and – 51.52 kcal/mol, respectively. These results were compared to free binding energy results of parent compounds NMJ-2 and NMJ-3, bio-isosteric structures showed better potency towards protein. Bio-isosteric structures were having a potential pharmacokinetic profile than parent compounds NMJ-2 and 3 (Table 6, 7, 8 and Fig. 4).

**Table 6:** XP-Docking score and free binding energy (MMGBSA) of bio-isosteric structures of NM-2 and NMJ-3

Compounds	Bio-isosteric structures	Docking score (XP)	MMGBSA Δ*G* bind (Kcal/mol)
NMJ-2	Structure-1	– 5.417	– 58.27
	Structure-2	– 5.376	– 57.12
	Structure-3	– 5.473	– 56.98
NMJ-3	Structure-1	– 5.876	– 53.12
	Structure-2	– 5.426	– 52.81
	Structure-3	– 5.044	– 51.52

**Table 7:**
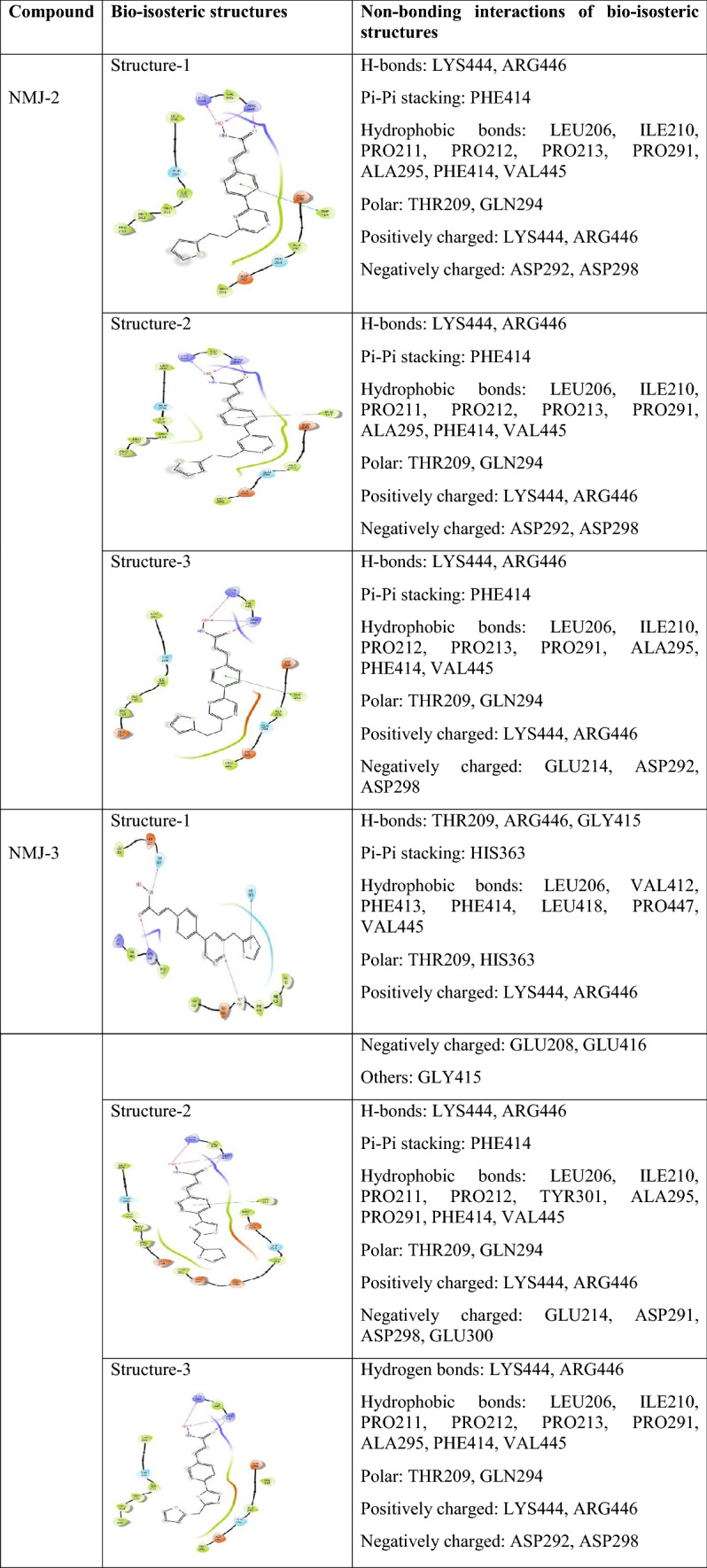
Non-bonding interactions of bio-isosteric structures of NMJ-2 and 3 during XP-docking

**Table 8:** Prediction of pharmacokinetic values (ADME) of bio-isosteric structures of NMJ-2 and NMJ-3 using QIKPROP tool

Compound	Compound Id	Molecular weight (MW)	QlogP_o/w_	Qlog HERG	QPlogS (mol.dm^−3^	QPPCaco	QPlogBB	% Human oral absorption	PSA	Rule of five (RO5)
Recommended values		130.0–725.0	2.0–6.5	< – 5	– 6.5 to – 0.5	< 25 Poor > 500 high	– 3.0 to 1.2 (inactive to active)	> 80% high < 25% poor	7.0–200.0	Max 4
NMJ-2	Structure -1	351.422	2.882	– 6.62	– 4.77	239.90	– 1.623	86.415	87.16	0
	Structure-2	351.422	2.739	– 6.56	– 4.84	157.02	– 1.841	82.285	94.23	0
	Structure-3	351.422	3.002	– 6.52	– 4.76	268.04	– 1.593	87.981	89.17	0
NMJ-3	Structure-1	321.335	1.837	– 6.30	– 3.86	142.68	– 1.841	76.293	101.79	0
	Structure-2	310.309	1.963	– 6.14	– 3.64	358.83	– 1.306	84.169	94.82	0
	Structure-3	311.296	1.514	– 6.21	– 3.72	139.38	– 1.793	74.189	110.89	0

### In-vivo study results

#### Anti-oxidant parameters

HFD + CCl4 significantly (*p* < 0.05) decreased the catalase, GSH, SOD, and nitrite levels when compared to the normal control group, which was significantly (*p* < 0.05) reversed by the oral administration of NMJ-3 and Sod.Val against NAFLD. On the other hand, HFD + CCl4 significantly (*p* < 0.05) enhanced levels of LPO in the NAFLD group when compared to the normal control. However, NMJ-3 at 50 mg/kg dose and Sod. Val at 25 mg/kg dose significantly (*p* < 0.05) increased levels of LPO in the treatment group than disease control (Table 9).

**Table 9:** Estimation of anti-oxidant parameters

Group	Catalase (units/mg of protein)	SOD (Units/mg of protein)	GSH (µM/mg protein)	Nitrite (ng/mg protein)	LPO (nM Malondialdehyde/mg protein)
Normal control	16.40 ± 2.780	20.65 ± 3.892	54.91 ± 3.594	28.85 ± 4.188	256.8 ± 29.79
Disease control	4.099 ± 0.4060**	7.136 ± 0.6225*	22.97 ± 0.3306***	12.21 ± 1.467**	650.2 ± 53.95**
Treatment (NMJ-3)	15.04 ± 1.408	18.65 ± 1.276^**#**^	40.20 ± 0.6307^**##**^	24.41 ± 0.5259^**#**^	367.4 ± 33.08^**#**^
Sod. Valproate	8.933 ± 0.7081^**#**^	20.39 ± 2.610^**#**^	38.11 ± 0.4397^**##**^	20.57 ± 0.7737	337.8 ± 51.01^**##**^

Anti-oxidant parameters represented in mean ± SEM

*p < 0.05 vs normal control

**p < 0.01 vs normal control

***p < 0.001 vs normal control

^#^*p* < 0.05 vs disease control

^##^*p* < 0.01 vs disease control.

### Biochemical parameters’ estimation

**Fig. 5:**
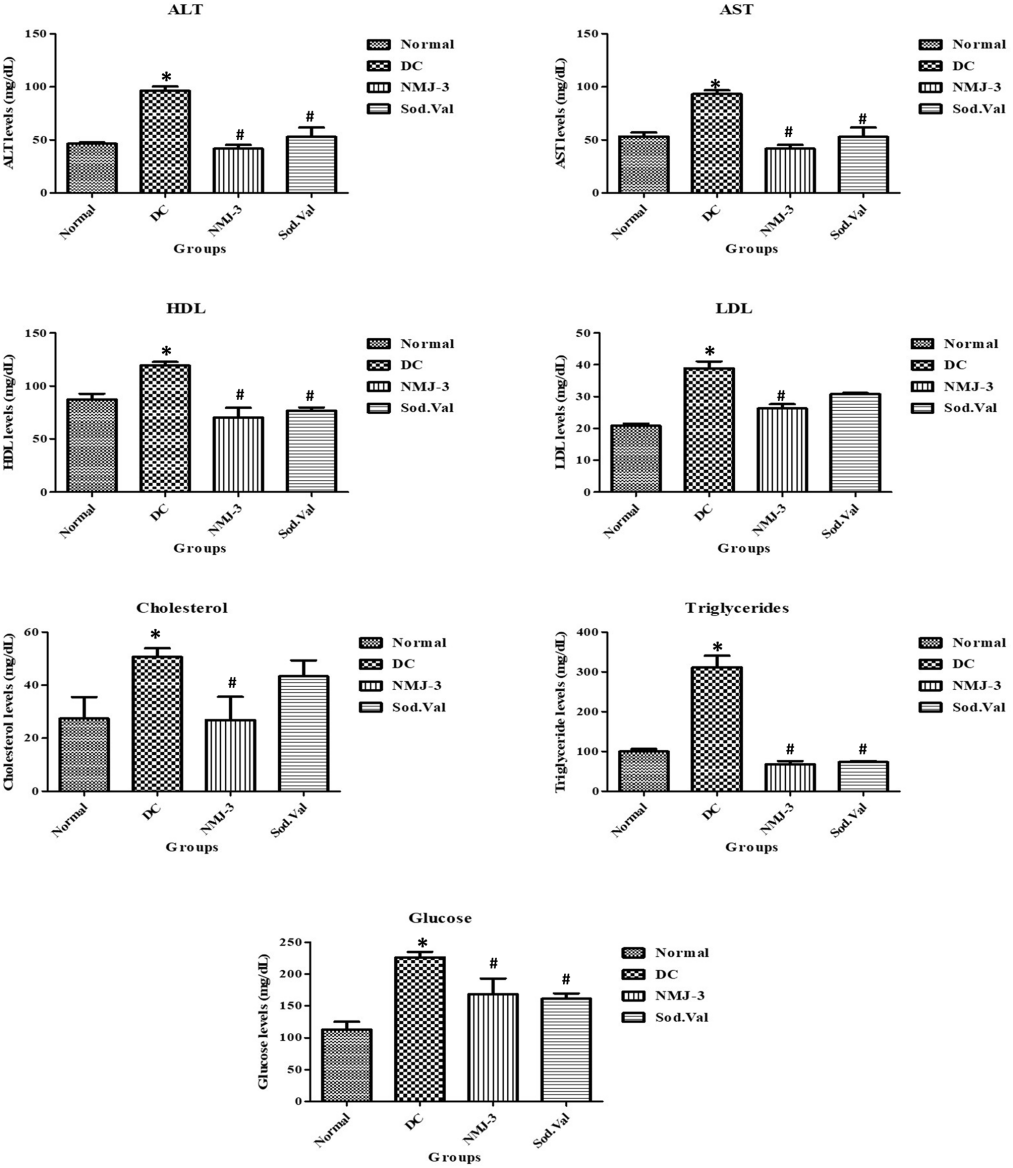
Graphical representation of biochemical parameters

Lipid profile parameters such as ALT, AST, HDL, LDL, cholesterol, TG, and glucose were significantly (*p* < 0.05) elevated in a diseased group compared to the normal control group. However, these results were downregulated significantly (*p* < 0.05) by the administration of NMJ-3 and SodiumValproate against NAFLD (Table 10 and Fig. 5).

**Table 10:** Estimation of biochemical parameters in plasma samples

Groups	ALT (IU/L)	AST (IU/L)	HDL (mg/dl)	LDL (mg/dl)	Cholesterol (mg/dl)	Triglyceride (mg/dl)	Glucose (mg/dl)
Normal control	46.27 ± 1.391	53.27 ± 3.755	87.28 ± 5.381	20.86 ± 0.6616	27.41 ± 8.136	99.68 ± 6.323	112.5 ± 12.93
Disease control	96.30 ± 4.140*	93.30 ± 3.554*	119.3 ± 3.375*	38.88 ± 2.211*	50.65 ± 3.272*	311.3 ± 29.53*	226.1 ± 8.806*
Treatment (NMJ-3)	41.74 ± 3.549^**#**^	41.74 ± 3.549^**#**^	70.38 ± 9.057^**#**^	26.24 ± 1.357^**#**^	26.93 ± 8.697^**#**^	67.80 ± 8.216^**#**^	168.7 ± 24.19^**#**^
Sodium Valproate	52.93 ± 8.716^**#**^	52.93 ± 8.716^**#**^	76.68 ± 3.010^**#**^	30.78 ± 0.4270	43.37 ± 5.976	73.19 ± 2.169^**#**^	161.4 ± 8.238^**#**^

Biochemical parameters’ comparison data represented in mean ± SEM

*p < 0.05 vs normal control

^#^*p* < 0.05 vs disease control

### Plasma insulin estimation

The disease group showed elevated levels of plasma insulin (*p* < 0.0001) that were found significant in comparison with the normal control group. A significant decrease was observed in both NMJ-3 and Sod. Val. treated groups (*p* < 0.0001) when compared to the disease group (Table 11).

**Table 11:** Estimation of plasma insulin

Groups	Normal control	Disease control	Treatment (NMJ-3)	Sod. Valproate
Plasma insulin (ug/ml)	3.70 ± 0.281	7.72 ± 0.405^****^	3.89 ± 0.302^####^	2.77 ± 0.144^####^

Plasma insulin levels are represented in mean ± SEM

****p < 0.0001 vs normal control

^####^*p* < 0.0001 vs disease control

### Histopathology of liver

**Fig. 6:**
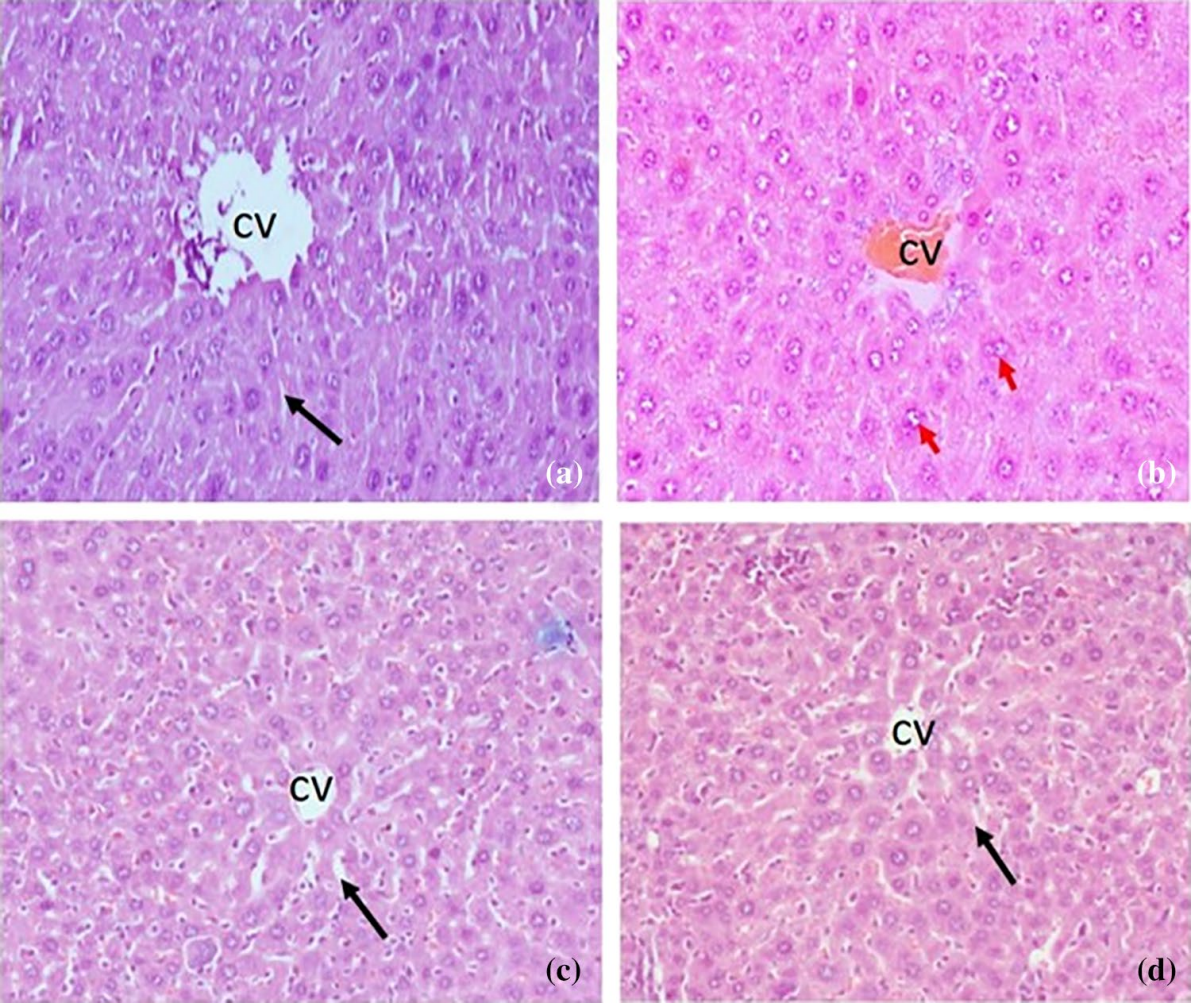
Histology of liver samples. Histology of HFD + CCl4 induced NAFLD in mice. Photomicrograph showing H and E section of mice liver (10X): Where **a** Normal control, **c** Disease control (DC), **b** Sodium valproate (SV), and **d** Treatment drug (NMJ-3), CV- central vein, black arrow—sinusoid, and red arrow- large cell changes of hepatocyte; **a** Shows normal control, with normal liver architecture; **c** Shows well-maintained liver architecture, with large cell changes in hepatocytes, sinusoidal congestion with mild microvesicular changes. Signs of inflammation, fibrosis, and necrosis are observed; **b** and **d** Shows well-restored liver architecture, with few large cell changes in the hepatocyte. No signs of inflammation, fibrosis, and necrosis are observed

Histology of NAFLD mice liver showed increased cell size and inflammation than normal control (NC). Standard control sodium valproate showed a reduction of inflammation and well-maintained liver architecture compared with NAFLD control. Treatment with NMJ-3 showed a decrease in inflammation; however, inflammation and any other liver changes were not observed in treatment groups (Fig. 6).

### Liver weight

After 14 days of treatment, animals were sacrificed and liver tissues were isolated and weighed. The change in liver weight was not significant across the groups.

### Discussion

NAFLD is one of the chronic metabolic disorders affecting liver histology that leads to a liver disease called NASH. It completely alters fatty acid metabolism and is stored in the form of TG and FFAs in hepatocytes. The severity of NASH causes hepatocellular carcinoma (HCC) (Liang et al. 2018). Alternative drugs have been used for treating NASH, but till now no drug has been approved exclusively for NASH. The current study was designed to check the effect of cinnamyl sulfonamide hydroxamate derivatives for treating NASH.

NOTCH-1 receptors are overexpressed in NAFLD/NASH conditions, which leads to insulin resistance by binding to the FoXO1 gene (Pajvani et al. 2011; Valenti et al. 2013; Zhao et al. 2018). Resveratrol as an SIRT-1 activator activated the SIRT-1 gene which in turn deacetylates the NOTCH-1 receptors (Zhao et al. 2013; Cao et al. 2015). In this study, cinnamyl sulfonamide hydroxamate derivatives were used for docking with 5BTR protein for activating the SIRT-1 gene. After ligand docking with protein, docking score, binding energy was compared with standard SIRT-1 activator resveratrol. They showed effective binding energy and good interactions with the target binding pocket in 5BTR protein. Based on results, cinnamyl sulfonamide hydroxamate derivatives were correlated with standard resveratrol results.

During molecular dynamic simulation analysis against vorinostat, all the interactions were lost that were seen in IFD-SP. AG446 was the strongest bond containing 60% simulation within the selected 100 ns trajectory period during MD analysis. NMJ-2 formed new interactions such as hydrogen bond at negatively charged ASP298, polar residue GLN294. This confirmation of the stability and structure formation of protein is due to the special characteristics of the side chain. Comparison of NMJ-3 results of XP-docking and induced-fit docking (IFD-SP), ASP298, GLU214, THR209, and ILE210 were present in the XP-docking interaction and IFD-SP contacts, except GLU214 not present in the IFD-SP. ASP298 was also present in the MD simulation of NMJ-2 and NMJ-3. This amino acid residue is necessary for the activity of the compound, and due to this, NMJ-3 was selected for further analysis on higher studies in in-vivo. NMJ-2 was not stabilized at end of the 100 ns trajectory period which explains the conformational changes that increase the probability of electrostatic interactions, which ensures more stability. NMJ-5 results showed stronger induced-fit interactions by losing all the low-energy interactions during docking, but in the 100 ns trajectory period of MD, it lost all XP interactions and IFD-SP interactions. It explains that the NMJ-5 was not docked well into the protein pocket and was not suitable for biological studies. Hence, hydrogen bonds are necessary for drug design, because they strongly influence the specificity of drugs, metabolism, and absorption.

Pharmacokinetic (ADME) values of cinnamyl sulfonamide hydroxamate derivatives along with vorinostat results were within the range compared with normal values. QPlogHERG for vorinostat, NMJ-6, NMJ-7 were between – 2 and – 6, which indicated that they might produce cardiac toxicity by blocking of K^+^ channel. These findings also highlighted that the synthesized drugs can be less toxic than vorinostat in terms of cardiotoxicity. The drug showed QPlogS in the range of – 3.6 to – 0.182, and the range of QPPCaco was 30 to 488.45. The QPlogBB was within – 2.1 to – 0.6 indicating less blood–brain barrier permeability. The percentage of human oral absorption was within 54 to 78. The PSA (oral bioavailability) of hydroxamate derivatives ranged from 65 to 144. All hydroxamate derivatives accepted Lipinski’s rule of five (RO5) and ligands were acceptable for oral absorption.

A high-fat diet (HFD) with CCl_4_-induced NASH model in mice was used for the *in-vivo* study (Jump et al. 2018; Kubota et al. 2013). HFD was supplied for 1 month and two doses of CCl_4_ were administered. CCl_4_ induces oxidative stress in hepatocytes leading to inflammation by producing reactive oxygen species (ROS). HFD causes insulin resistance (IR) in disease-induced groups, and HFD + CCl_4_ increases chronic liver inflammation and liver injury. All biochemical parameters like cholesterol, TG, AST, ALT, HDL, LDL, and glucose were estimated to check whether the disease was inducted. All the parameters showed a significant (*p* < 0.05) increase in the disease-induced group when compared with normal control. Treatment with NMJ-3 significantly reversed the changes and it was in sync with *in-silico* results. Effective *in-vivo* activation of SIRT-1 by NMJ-3 inhibited NOTCH-1 receptors and successfully decreased the disease progression. This also significantly reduced plasma insulin level as insulin resistance was nullified. NOTCH signalling is positively correlated with insulin resistance. High NOTCH signalling increases insulin resistance and hyperglycemia (Khan et al. 2016). Effective inhibition of this signalling due to NMJ-3 treatment significantly reduced the insulin levels by reducing insulin resistance.

## Conclusion

The combination of HFD and CCl_4_ altered the lipid metabolic functions which led to the storage of cholesterol and triglycerides in hepatocytes in CF-1 male mice. In contrast, hydroxamate derivative NMJ-3 (50 mg/kg) and sod. valproate (25 mg/kg) reduced the storage of lipid content in hepatocytes in the in-vivo model as predicted in in-silico studies. A detailed study of the above results concluded that NMJ-2 and NMJ-3 can inhibit the NOTCH-1 overexpression by activation of SIRT-1 in NAFLD/NASH in comparison with vorinostat. Further higher studies are required for molecular-level confirmation.

## Abbreviations

ADME: Absorption, Distribution, Metabolism, Excretion
CD: C-terminal domain
FFA: Free fatty acids
GLIDE: Grid-based ligand docking with energetics
HCC: Hepatic cellular carcinoma
HFD: High-Fat Diet
IFD: Induced fir docking
IR: Insulin resistance
MD: Molecular dynamics
MM-GBSA: Molecular Mechanics/Generalized Born Surface Area
NAD: Nicotinamide adenine dinucleotide
NAFLD: Non-alcoholic fatty liver disease
NASH: Non-alcoholic steatohepatitis
NECD: Notch extracellular domain
NICD: Notch intracellular domain
NTD: N-terminal domain
PDB: Protein data bank
RMSD: Root-mean-square deviation
SP: Standard precision
SREBP1c: Sterol regulatory elementary binding protein 1 c
TG: Triglycerides
XP: Extra precision
